# From Behavioral and Sleep Disturbances to Genetic Diagnosis: Smith–Magenis Syndrome and the Importance of the Diagnostic Pathway

**DOI:** 10.1002/dneu.70051

**Published:** 2026-07-03

**Authors:** Fethiye Kılıçaslan, Ayşe Rümeysa Olgar Çetin, Özlem Öz, Mert Coşkun, Mehmet Burak Mutlu, Berna Polat Tüysüz

**Affiliations:** ^1^ Department of Child and Adolescent Psychiatry, Faculty of Medicine Harran University Sanliurfa Turkey; ^2^ Department of Medical Genetics, Faculty of Medicine Harran University Sanliurfa Turkey; ^3^ Department of Medical Genetics Sanliurfa Mehmet Akif İnan Education and Research Hospital Sanliurfa Turkey; ^4^ Department of Medical Genetics Detagen Genetic Diseases Evaluation Center Kayseri Turkey

**Keywords:** child psychiatry, genetic counseling, neurodevelopmental disorder, retinoic acid‐induced 1 (RAI1), sleep disorders, Smith–Magenis syndrome

## Abstract

Smith–Magenis syndrome (SMS) is a rare multisystem genetic disorder caused by a 17p11.2 microdeletion or pathogenic variants in the retinoic acid‐induced 1 (*RAI1*) gene. It is characterized by developmental delay, distinctive craniofacial features, behavioral dysregulation, and inverted sleep–wake rhythm. Because early clinical findings are often nonspecific, diagnosis is frequently delayed, and patients may initially present to child psychiatry services with behavioral complaints. We report a 5‐year‐old girl referred for hyperactivity, severe circadian sleep disturbance with recurrent nocturnal awakenings, self‐injurious behaviors, sensory‐seeking behaviors, and developmental delay. Comprehensive psychiatric, developmental, neurological, physical, and genetic evaluations revealed a 17p11.2 microdeletion involving both *RAI1* and *folliculin (FLCN)*. The diagnosis of SMS was confirmed, and the involvement of *FLCN* indicated additional potential long‐term medical risks. This case underscores the importance of considering genetic etiologies in children presenting with severe behavioral dysregulation and sleep problems and highlights the critical role of comprehensive genetic assessment in guiding diagnosis, management, and long‐term follow‐up.

## 1 Introduction

Smith–Magenis syndrome (SMS) is a rare, complex genetic disorder associated with a 17p11.2 microdeletion or pathogenic the retinoic acid‐induced 1 (*RAI1*) variants, with an estimated prevalence of 1 in 15,000–25,000 individuals (Rinaldi et al. 2022). Common behavioral manifestations include hyperactivity, impulsivity, self‐injurious behaviors, sensory‐seeking tendencies, sleep disturbance, and repetitive stereotypic behaviors that may resemble other neurodevelopmental disorders (Rinaldi et al. 2022). Nearly all affected individuals have circadian rhythm inversion, which worsens sleep and behavioral problems (Poisson et al. 2015).

Because early manifestations may initially resemble attention‐deficit/hyperactivity disorder (ADHD), autism spectrum disorder (ASD), or isolated developmental delay, and the full syndromic phenotype may emerge gradually over time, diagnosis is often delayed until genetic confirmation is obtained. Children may therefore first present to child psychiatry services with hyperactivity, sleep disturbance, self‐injurious behavior, or developmental concerns rather than with suspicion of an underlying genetic syndrome (Rinaldi et al. 2022; Laje et al. 2010; Orpay et al. 2024; Khan and Pradhan 2019). Early recognition is clinically important because delayed diagnosis may postpone appropriate multidisciplinary surveillance, targeted management of sleep and behavioral problems, and genetic counseling for the family. In patients with larger 17p11.2 deletions, involvement of adjacent genes such as *folliculin (FLCN)* may also have clinical implications, particularly regarding long‐term surveillance for Birt–Hogg–Dubé syndrome–related manifestations (Schmidt and Linehan 2018). In this report, we describe a 5‐year‐old girl who presented with hyperactivity, severe sleep disturbance, and self‐injurious behaviors and was subsequently diagnosed with SMS following comprehensive clinical and genetic evaluation.

## 2 Methods

In this study, we present the clinical features and diagnostic process of a 5‐year‐old girl who was referred to the child and adolescent psychiatry outpatient clinic with complaints of severe behavioral dysregulation and prominent sleep disturbances. Written informed consent was obtained from the patient's family. The evaluation was conducted using a multidisciplinary approach, including a detailed psychiatric examination according to the Diagnostic and Statistical Manual of Mental Disorders, Fifth Edition (DSM‐5) criteria, developmental screening with the Ankara Developmental Screening Inventory (ADSI), a standardized caregiver‐based developmental screening tool widely used in Türkiye to assess language‐cognitive, fine motor, gross motor, and social self‐care developmental domains (Sezgin 2011), neurological assessment with cranial MRI, physical examination focusing on dysmorphic features, and cardiology and orthopedic consultations.

For etiological investigation, peripheral venous blood samples were obtained for chromosomal analysis and molecular karyotyping. Conventional karyotype analysis revealed a 46, XX result, and no numerical or gross structural chromosomal abnormalities were detected in the analyzed metaphases. Genomic DNA was isolated from peripheral blood using the QIAamp DNA Blood Mini Kit (Qiagen Inc., Basel, Switzerland). Molecular karyotyping was performed using Infinium Global Screening Array Cyto (GSA‐Cyto) microarray chips.

### 2.1 Data Analysis

The obtained data were analyzed using the BlueFuse Multi 4.5 software in conjunction with up‐to‐date databases, including PubMed, OMIM, Database of Genomic Variants (DGV), and DECIPHER. For copy number variation (CNV) analysis, population frequency data were assessed using the Genome Aggregation Database (gnomAD) and the DGV. To identify individuals with similar structural variants, define the gene regions encompassed by the structural alteration, and determine the genomic distances between these regions, the Database of Genomic Variation and Phenotype in Humans using Ensembl Resources (DECIPHER) was utilized. Variant classification was performed in accordance with the variant interpretation guidelines published by the American College of Medical Genetics and Genomics (ACMG, 2015) and the Association for Clinical Genomic Science (ACGS, 2019) (Ellard et al. 2019; Richards et al. 2015).

## 3 Results

A 5‐year‐old girl was brought to the child psychiatry outpatient clinic by her mother due to hyperactivity, markedly irregular sleep, self‐biting, head banging against walls, persistent mouthing of objects, and removal of her diaper at night to manipulate feces.

According to her mother, the family first became concerned about her development at around 2 years of age, when the patient had not yet achieved independent walking and showed delayed language development. The family subsequently sought evaluation at a pediatric neurology clinic. Developmental history indicated that she spoke her first words at 2 years of age, achieved independent ambulation at 4 years with physiotherapy support, and formed two‐word combinations at 5 years; however, expressive language remained limited and poorly intelligible. Toilet training had not been completed, and she received special education services once weekly. Pregnancy had been uneventful with regular prenatal follow‐up, and delivery occurred at term via spontaneous vaginal birth.

The family also reported that sleep disturbances became particularly prominent around 3 years of age and progressively worsened over time. According to the caregiver, the child routinely awakened between 02:00 and 03:00 and was unable to resume sleep; during the daytime, she exhibited two brief sleep episodes lasting approximately 1 h each. Behavioral concerns, including hyperactivity, self‐injurious behaviors, and aggression toward peers, also became prominent during the preschool period; however, the exact age of onset could not be determined reliably from retrospective caregiver history. Aggression toward peers was described in the context of behavioral dysregulation and frustration intolerance; however, the caregiver history did not allow reliable differentiation between impulsive, sensory‐related, or unprovoked episodes. She had reportedly been denied admission to a special education and training school due to aggressive behaviors toward peers.

On psychiatric examination, the patient appeared calm and appropriately groomed. Eye contact and joint attention were adequate. She responded to her name when called and did not exhibit marked impairment in reciprocal social interaction. She followed simple verbal commands and produced scribbles using crayons placed on the table. Her attention span was limited, and her level of motor activity exceeded expectations for her chronological age and developmental status. During the examination, stereotypic self‐hugging behavior and repetitive lateral head‐turning movements were observed. The self‐hugging behavior was considered a clinically relevant diagnostic clue supporting the suspicion of SMS. Her voice was deep and hoarse, and she produced short utterances; her speech was intelligible only to her mother.

**FIGURE 1:**
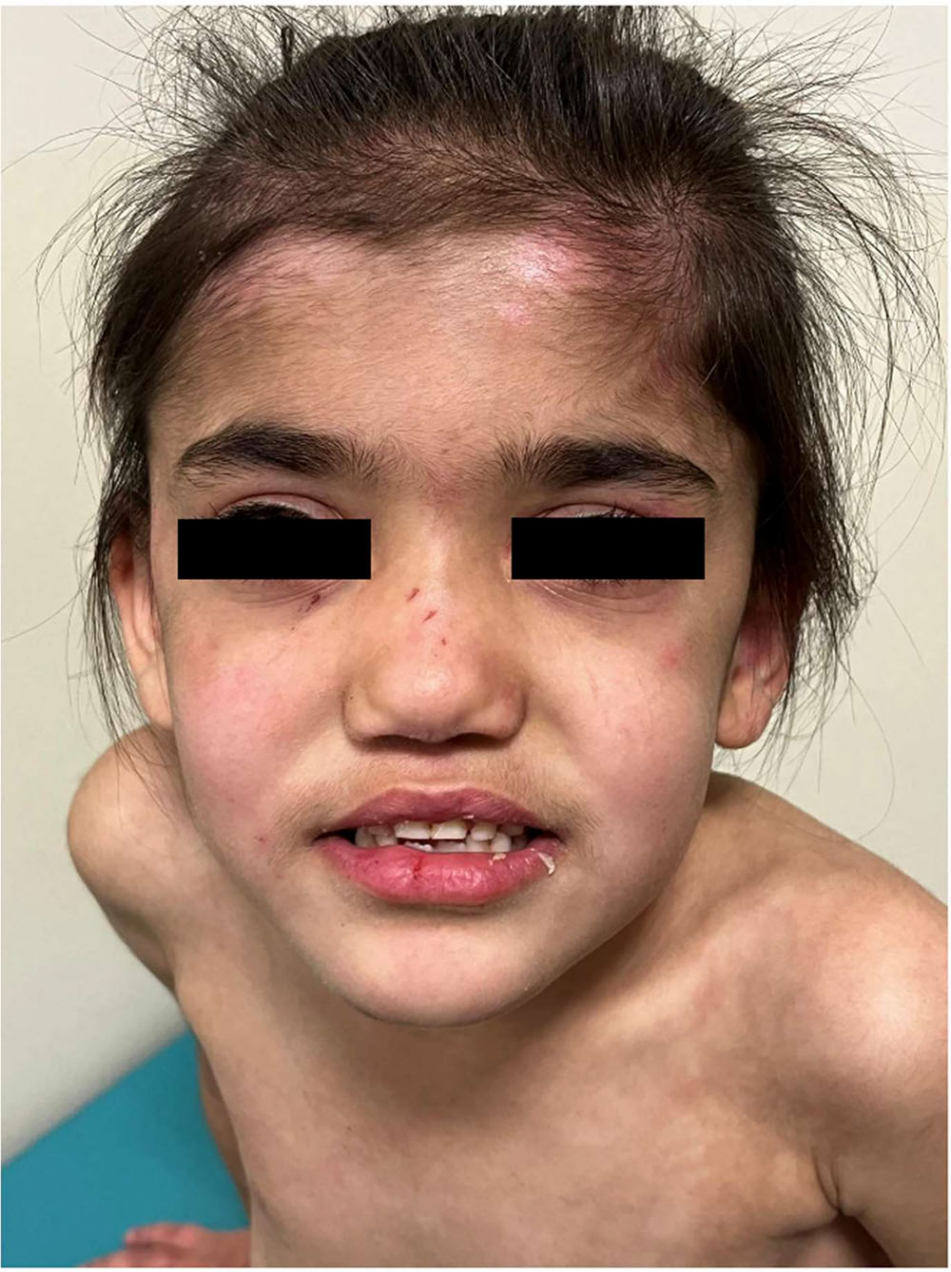
Phenotypic features of the patient.

Physical examination revealed a syndromic facial phenotype characterized by triangular and coarse facial features, prominent supraorbital ridges, long palpebral fissures, long eyelashes, prominent nasal alae, a short philtrum, and a tented upper lip. Mild brachydactyly was also noted (Figure 1). Developmental assessment using the ADSI indicated a general developmental level corresponding to 35 months; language‐cognitive developmental age of 32 months; fine motor developmental age of 36 months; gross motor developmental age of 45 months; and social skills–self‐care developmental age of 27 months. Family history revealed no parental consanguinity and no similarly affected relatives.

The patient had been followed by pediatric neurology due to congenital hypotonia and developmental delay. Cranial magnetic resonance imaging demonstrated a mega cisterna magna, and cardiological evaluation identified a secundum atrial septal defect. Orthopedic assessment documented lower extremity curvature, requiring surgical planning and follow‐up. On the basis of the DSM‐5 criteria, she met diagnostic thresholds for a mild delay in cognitive development, ADHD, and sleep disorder.

Initial management included sleep hygiene recommendations and behavioral sleep strategies, such as establishment of a consistent bedtime routine and reduction of nighttime environmental stimulation. Aripiprazole 2 mg/day was then initiated to address severe behavioral dysregulation and associated sleep disturbance, and outpatient follow‐up was recommended. Given the constellation of findings suggestive of a syndromic condition, she was referred to medical genetics for further evaluation. A formal audiological assessment was planned due to reported selective responsiveness to high‐intensity auditory stimuli.

Approximately 1 year later, she returned to the clinic. Her mother reported that recommended outpatient follow‐up had not been maintained. According to caregiver report, aripiprazole 2 mg/day had been used inconsistently and was later discontinued by the family without medical consultation. Objective pharmacy refill or adherence data were not available. Sleep disturbance and self‐injurious behaviors persisted.

**FIGURE 2:**
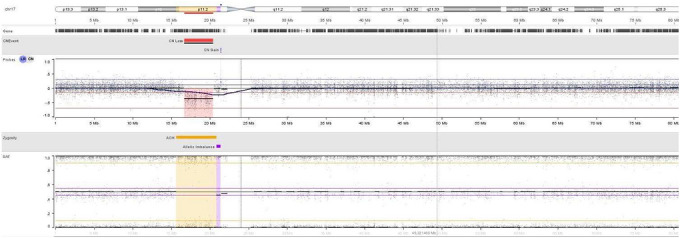
Chromosomal microarray analysis image: a heterozygous deletion of approximately 3.7 Mb was detected in the 17p11.2 region in the patient.

Previously requested genetic analyses were reviewed. Conventional karyotyping revealed a normal female complement (46, XX). Chromosomal microarray analysis revealed a heterozygous deletion of approximately 3.7 Mb at the 17p11.2 region. This region includes the *RAI1* gene (Figure 2). According to OMIM, deletions involving this region are associated with the phenotype of SMS. The detected variant has not been reported in healthy population studies. Following a review of the current literature and database analyses, this alteration was classified as a “pathogenic” CNV. The deletion also encompassed the *FLCN* gene, which is associated with Birt–Hogg–Dubé syndrome. Parental carrier testing was recommended to the family to determine whether the mutation was de novo.

Due to persistent nocturnal awakenings and circadian rhythm disturbance, melatonin 3 mg at bedtime was initiated. However, the response to melatonin monotherapy was insufficient, and low‐dose risperidone solution 0.25 mg/day was subsequently added. No formal polysomnographic sleep study or actigraphy was performed; sleep disturbance was assessed clinically on the basis of caregiver report. According to the caregiver's report, combined treatment with melatonin and risperidone was associated with marked improvement in sleep regulation and behavioral symptoms. The exact time to improvement after treatment initiation could not be reliably determined because of inconsistent follow‐up and retrospective caregiver reporting. The overall clinical picture was considered consistent with SMS and was characterized by mild delay in cognitive development, ADHD, severe behavioral dysregulation, circadian rhythm disturbance, sensory‐seeking behaviors, and speech impairment.

## 4 Discussion

In many patients with SMS, behavioral symptoms dominate the early clinical picture and may lead to psychiatric referral before the underlying genetic syndrome is recognized (Rinaldi et al. 2022). The present case highlights the diagnostic challenge of distinguishing SMS from primary psychiatric or neurodevelopmental conditions. Although the patient presented at an age when SMS may be clinically recognized, the initial manifestations were relatively nonspecific and evolved gradually over time. Hyperactivity, developmental delay, self‐injury, and severe sleep disturbance were initially considered within a broader neurodevelopmental framework. However, the progression from early developmental concerns to increasingly prominent circadian sleep disturbance and behavioral dysregulation was broadly consistent with the behavioral trajectory described in SMS. The coexistence of characteristic behavioral features, dysmorphic findings, circadian sleep–wake disturbance, and multisystem involvement therefore prompted genetic evaluation, which revealed a 17p11.2 deletion involving *RAI1* and *FLCN*. This finding confirmed the diagnosis of SMS as well as indicated the need for long‐term surveillance related to *FLCN* involvement. Thus, the diagnostic trajectory in this case reflects the clinical complexity of SMS and underscores the importance of considering syndromic etiologies in children presenting with behavioral dysregulation, developmental delay, and severe sleep disturbance.

Neurological manifestations of SMS include infantile hypotonia, hyporeflexia, global developmental delay, peripheral neuropathy findings, structural central nervous system variations, and epileptic seizures (Rinaldi et al. 2022). The congenital hypotonia and marked motor delay observed in this patient are consistent with the established neurological spectrum of the disorder. The mega cisterna magna identified during neurological follow‐up represents a nonspecific structural variation of the central nervous system previously reported in SMS (Rinaldi et al. 2022). Although epileptic seizures were not observed in this case, the documented association of epilepsy and subclinical EEG abnormalities in SMS highlights the importance of ongoing neurological surveillance.

A defining feature of SMS is its distinctive behavioral phenotype. This phenotype encompasses inverted circadian rhythm, severe sleep–wake dysregulation, self‐injurious behaviors, sensory‐seeking tendencies, impulsivity, and syndrome‐specific stereotypies (Rinaldi et al. 2022). The pronounced nocturnal awakenings and fragmented sleep architecture in this patient are consistent with the well‐documented abnormalities in melatonin secretion in SMS (Smith et al. 2019; Potocki et al. 2000). Behaviorally, the presence of repetitive motor stereotypies in addition to self‐injury is particularly noteworthy. The observed repetitive lateral head‐turning movements and characteristic self‐hugging behavior align with stereotypic motor patterns described as relatively specific to SMS (Martin et al. 2006). Self‐hugging, in particular, has been highlighted in the literature as a distinctive behavioral marker and may serve as a valuable diagnostic clue in clinical practice.

The prominent oral sensory‐seeking behavior—manifested by persistent placement of objects in the mouth—may reflect underlying sensorimotor integration and regulatory difficulties. Altered sensory processing and sensory‐seeking profiles are frequently reported in SMS and may exacerbate both self‐injurious behaviors and impairments in adaptive functioning (Martin et al. 2006). The patient's limited, hoarse, and caregiver‐dependent speech intelligibility is consistent with the SMS speech‐language phenotype, characterized by delayed expressive language development and persistent articulation difficulties (Brennan et al. 2024). Collectively, the constellation of behavioral and neurodevelopmental features in this case clearly exemplifies the prototypical behavioral phenotype of SMS.

SMS is a multisystem disorder affecting growth, musculoskeletal development, otolaryngological structures, vision, cardiovascular integrity, and immune function (Rinaldi et al. 2022). Hearing loss and hyperacusis are among the most commonly reported features. Laryngeal and velopharyngeal anomalies frequently result in a deep, hoarse, and muffled voice quality (Brennan et al. 2024). Although a formal audiological assessment could not be completed due to a middle ear infection, the patient's selective responsiveness to high‐intensity auditory stimuli and characteristic voice quality are consistent with previously described otolaryngological manifestations of SMS. Ocular findings may include strabismus, progressive myopia, and iris anomalies. Peripheral neuropathy‐associated lower extremity deformities, gait abnormalities, and early childhood growth delay are also well documented (Rinaldi et al. 2022). Oral–dental anomalies and obesity may variably accompany the phenotype (Tomona et al. 2006; Elatrash et al. 2024), and hypercholesterolemia independent of dietary factors and body mass index has been identified as a metabolic feature of SMS (Elatrash et al. 2024; Smith et al. 2002). Congenital heart defects, including atrial septal defect, have likewise been reported (Onesimo et al. 2021). The short stature, lower extremity deformity requiring surgical planning, and secundum atrial septal defect observed in this patient are therefore consistent with the recognized multisystem involvement of SMS.

From a differential diagnostic perspective, SMS should be considered within the broader spectrum of genetic syndromes and neurodevelopmental disorders characterized by developmental delay, hypotonia, and severe behavioral dysregulation. Angelman and Prader–Willi syndromes are often initially considered due to overlapping early clinical features (Cassidy et al. 2012). However, the frequent occurrence of epilepsy and microcephaly in Angelman syndrome and childhood‐onset hyperphagia with progressive obesity in Prader–Willi syndrome provides distinguishing features (Cassidy et al. 2012). Other genetic conditions—including 22q11.2 deletion syndrome, Kleefstra syndrome, White–Sutton syndrome, *MBD5* haploinsufficiency, Vulto‐van Silfout‐de Vries syndrome, *CHD2*‐ and *KAT6A*‐related neurodevelopmental disorders, and Fragile X syndrome—may also enter the differential diagnosis due to shared cognitive and behavioral characteristics (Rinaldi et al. 2022).

Clinical overlap with ASD is relevant because restricted/repetitive behaviors, sensory‐seeking behaviors, and social‐communication concerns may occur in both ASD and SMS (Laje et al. 2010; Potocki et al. 2000). In the present case, however, spontaneous eye contact, response to name, basic joint attention, and reciprocal engagement were relatively preserved for her developmental level, making a primary ASD presentation less likely. This distinction is clinically important, as preserved social reciprocity despite the presence of stereotypic and sensory‐seeking behaviors may serve as a useful clue against primary ASD in some patients with SMS. In contrast, the combination of circadian sleep–wake disturbance, self‐injurious behavior, self‐hugging, persistent mouthing of objects, dysmorphic features, hypotonia, and multisystem findings supported syndromic evaluation (Ziats et al. 2021; Kılıçaslan et al. 2025). Thus, when ASD‐like symptoms occur together with these additional features, genetic testing should be considered rather than attributing the presentation solely to a primary neurodevelopmental disorder.

The identification of a 17p11.2 microdeletion encompassing both the *RAI1* gene and the adjacent *FLCN* gene is clinically important. The patient's neurodevelopmental, behavioral, sleep‐related, and dysmorphic features were considered most consistent with the established phenotype of SMS, primarily related to *RAI1* haploinsufficiency. Although the deletion also included *FLCN*, no causal relationship between *FLCN* haploinsufficiency and the patient's neuropsychiatric symptoms can be inferred from this single case. The clinical significance of *FLCN* in this context is mainly related to its established association with Birt–Hogg–Dubé syndrome, which is primarily characterized by adult‐onset cutaneous, pulmonary, and renal manifestations (Toro et al. 2008). Therefore, the coexistence of *RAI1* and *FLCN* deletions warrants cautious phenotypic interpretation, comprehensive genomic evaluation, genetic counseling, and long‐term multidisciplinary surveillance, including renal imaging and monitoring for pulmonary manifestations related to *FLCN* involvement.

The pharmacological approach in the present case was guided by the patient's dominant clinical problems, particularly severe circadian sleep disturbance, behavioral dysregulation, aggression, and self‐injurious behaviors. Sleep disturbance in SMS is closely related to disruption and inversion of melatonin secretion, and management commonly includes sleep hygiene strategies together with evening melatonin or other sleep‐promoting agents. Improvement in sleep may also contribute to better behavioral regulation and caregiver quality of life. In this context, melatonin was selected to target the prominent nocturnal awakenings and circadian sleep–wake disturbance (Kaplan et al. 2020). Aripiprazole and later low‐dose risperidone were used symptomatically to address severe behavioral dysregulation, irritability, aggression, and self‐injurious behaviors. Although there are no standardized pharmacological guidelines for behavioral problems in SMS, previous literature emphasizes that treatment should be individualized, symptom‐targeted, and integrated with behavioral, developmental, family‐based, and multidisciplinary interventions (Poisson et al. 2015).

This case report has several limitations. First, some early developmental and behavioral information was obtained retrospectively from caregiver report, and the exact age at onset of behavioral symptoms could not be determined with certainty. Although developmental functioning was assessed using the ADSI, no formal cognitive test was performed; therefore, intellectual functioning could not be characterized in greater detail. Second, no formal polysomnographic sleep study or actigraphy was performed; therefore, the characterization of circadian sleep disturbance and treatment response was based on clinical history and caregiver report rather than objective sleep parameters. Third, inconsistent follow‐up and irregular medication adherence during the early course limited assessment of the longitudinal clinical trajectory and treatment response.

## 5 Conclusion

This case highlights that severe sleep disturbance with circadian inversion, particularly when accompanied by self‐injurious behavior, developmental delay, behavioral dysregulation, dysmorphic features, hypotonia, or multisystem involvement, should raise clinical suspicion for SMS and prompt genetic evaluation. Because such patients may initially present to child psychiatry services with apparently nonspecific developmental and behavioral symptoms, child psychiatrists have an important role in recognizing syndromic red flags and coordinating timely referral for genetic and multidisciplinary evaluation. Establishing a genetic diagnosis can guide not only immediate behavioral and developmental management but also anticipatory guidance, genetic counseling, and long‐term medical surveillance. In cases involving contiguous gene deletions, careful interpretation of all genes within the deleted interval is essential, as additional genes such as *FLCN* may have lifelong surveillance implications beyond the primary SMS phenotype. Early recognition and multidisciplinary intervention are critical for optimizing clinical management, coordinating surveillance, and supporting affected children and their families.

## Author Contributions

Conceptualization: Fethiye Kılıçaslan and Berna Polat Tüysüz. Data curation: Fethiye Kılıçaslan, Ayşe Rümeysa Olgar Çetin, Özlem Öz, Mert Coşkun, and Mehmet Burak Mutlu. Investigation: Fethiye Kılıçaslan, Ayşe Rümeysa Olgar Çetin, Özlem Öz, Mert Coşkun, and Mehmet Burak Mutlu. Formal analysis: Özlem Öz, Mert Coşkun, and Mehmet Burak Mutlu. Methodology: Fethiye Kılıçaslan, Özlem Öz, Mert Coşkun, and Mehmet Burak Mutlu. Project administration: Fethiye Kılıçaslan. Resources: Fethiye Kılıçaslan, Özlem Öz, Mert Coşkun, Mehmet Burak Mutlu, and Berna Polat Tüysüz. Supervision: Berna Polat Tüysüz. Validation: Fethiye Kılıçaslan, Özlem Öz, Mert Coşkun, Mehmet Burak Mutlu, and Berna Polat Tüysüz. Writing – original draft: Fethiye Kılıçaslan and Ayşe Rümeysa Olgar Çetin. Writing – review and editing: Fethiye Kılıçaslan, Ayşe Rümeysa Olgar Çetin, Özlem Öz, Mert Coşkun, Mehmet Burak Mutlu, and Berna Polat Tüysüz. All authors read and approved the final version of the manuscript.

## Funding

The authors have nothing to report.

## Ethics Statement

All procedures in the study were performed according to the World Medical Association Declaration of Helsinki.

## Consent

Informed consent was obtained from the patient and/or their legal guardian for participation in this report.

## Conflicts of Interest

The authors declare no conflicts of interest.

## Data Availability

The datasets used and/or analyzed during the current study are available from the corresponding author on reasonable request.
